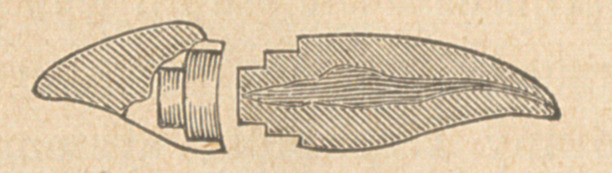# Central Dental Association of Northern New Jersey

**Published:** 1883-07

**Authors:** James G. Palmer

**Affiliations:** Secretary


### THE CENTRAL DENTAL ASSOCIATION OF NORTHERN NEW JERSEY.

The regular monthly meeting for the month of May, was called to order on Thursday Evening, May 31st, at the office of Dr. C. A. Timme, 190 Hudson Street, Hoboken, N. J. President Levy in the chair.

After the usual routine business, election of members and reports of committees, the following paper was read by Dr. James G. Palmer, of New Brunswick, on "Treatment of Devitalized Teeth."

*Mr. President and Gentlemen of the Central Dental Association of Northern New Jersey:*

At our March meeting, President Levy, without so much as by your leave, said, "Dr. Marsh will write a paper for our April meeting, and Dr. Palmer, for our May meeting." I did not feel much in the humor for it until the following incident occurred in my office practice. A young man, about twenty years of age, came to me and desired me to do whatever work might be needed in his mouth. I found the right superior central dead, and the fillings on both approximal surfaces in need of attention, one being gutta percha. I made some inquiry concerning the tooth, and learned that the patient had received a severe blow upon the face about three years before; that the tooth had been very sore and the face much swollen. The tooth was subsequently filled by a dentist whose name he mentioned. Being acquainted with the dentist, I wrote him concerning it, and was astonished upon receiving his reply. He said he did not think that he had treated the tooth, as he had nearly given up practice, and "never treated any but the six fronts, upper," seldom treating these now. Why, he did not say, but I was surprised, because from my knowledge of the gentleman I presumed that he followed a different line of practice. I removed the filling, found the pulp chamber full of gutta-percha, which I also removed, and found beyond it a little cotton, a short piece of steel (probably a broach) and a great deal of dark debris which gave forth an unmistakable and disagreeable odor.

With this incident as my incentive, I take the "Treatment of

Devitalized Teeth" as my text, and divide the subject in the following manner:

1st. Treatment when the nerve has been recently devitalized.

2d. Treatment, as in the case mentioned, when the tooth has been dead for some time, but the gums are in an apparently healthy condition; and

3d. Treatment in cases where an abscess exists in connection with the tooth, either closed, or discharging through a fistula.

First, let me describe some instruments that I prize highly for this work. I do not like to use the ordinary barbed broach, such as we usually find at the dental depots. Perhaps it is because I was taught differently, and feel inclined to follow that line of practice which in other hands has proven so successful. I obtain what are known as Swiss Pivot Broaches. Jewelers use them for reaming out the little holes in their fine work. I generally get as fine as 0 or 00. They are hard temper, so hard that they break very readily, and will burn if put at once into a flame, but by drawing the temper carefully they can readily be bent in any direction, and will follow a very crooked canal. In connection with these I use unwaxed dental floss silk, which possesses the advantage over cotton of giving a long, delicate fibre, which is not easily broken. This I wind around the broach, when it is ready for use. As these broaches are four-sided, silk is readily attached to them. I prefer to use carbolic acid crystals rather than creasote.

To refer to the treatment:

When the nerve has recently been destroyed, no matter how, I use one of these smooth four or five-sided broaches with this delicate wisp of floss silk upon it, gently insinuate it up the canal as far as I can, twist it dexterously, when the floss silk will entangle the nerve and wrap it around the broach, and, in a majority of instances, bring it out entire. The bleeding that follows will soon cease. I then use a number of these broaches with more or less silk wound on them, according to the size of the canal, to wipe out all moisture, and dressing with carbolic acid, seal it up tightly. I see the case in two or three days; usually it may be filled at this sitting, but in some instances I close it up the second time and fill at the third sitting. Very rarely do I find it necessary to make more than the third appointment. In cases where the broach and silk fail to bring out all the contents of the nerve canal, I use Donaldson's nerve instruments, and with the little hooked end endeavor to break up the portion remaining in the tooth. These cases are the ones apt to need a third sitting.

When the tooth has been devitalized for some time, as in the instance above mentioned, if the nerve remains in the tooth in an almost semi-liquid condition, really putrified, the broach and floss will invariably bring it out intact, while the barbed broach will often break it into pieces. I use the broaches in the same manner as before to cleanse out the canal thoroughly, taking care not to go through the apicial foramen. It may be necessary to use a great many broaches, but 1 find that they cleanse the canal more thoroughly than bibulous paper, when twisted into strings, can possibly do. Having thoroughly cleansed the canal, I use a broach with floss silk as a piston, and pump some dilute carbolic acid up the canal. Usually I dilute with glycerine, as it has wonderful penetrating properties, and will carry the carbolic acid well up the tooth canal. I then close up the canal with cotton, loosely packed. In this division I spoke of the gums being in an apparently healthy condition. The treatment above described may produce a state of affairs such as exists in the next division, but generally it will not, and if at the second sitting no trouble has been experienced I again cleanse, using the broaches and carbolic acid full strength, and close the canal tightly, usually sealing it with gutta percha. Sometimes it may be necessary to renew this dressing on several occasions; sometimes only a few sittings are needed. I am not now prepared to say whether we do not oftentimes treat a tooth too long. That question, I think, will admit of some close observation at least.

When an abscess exists, I prefer, if there is no fistulous opening, to make one, and then, cleansing the canal as before, to pump pure carbolic acid through the apex, or force it through with one of Farrar's syringes. If it comes out of the fistulous opening on the first attempt, the case is dismissed in a very few days. If it does not come through, it may take a little longer, but in either case I close up the canal tightly, and at the second or third sitting proceed to fill the canal, after which, if the fistula still exists, it may be healed by one or two injections of carbolic acid through the opening by means of a Farrar syringe, always providing there is no necrosed condition. That forms a subject by itself.

Having now reached the time for filling the canal, I use the broach with the floss silk again. Dr. Watkins ably described my method at our last meeting, which method is really not mine, but that which with Dr. Watkins and others, I heard Dr. McKellops, of St. Louis, describe some years ago. I use gutta percha dissolved in chloroform, either the pink of the laboratory, or Hill's stopping. Using the broach as a piston, this solution can be readily pumped up the canal to the apex. I have some rolls of gutta percha, about the size of a broom splint or fine broach, prepared and cut into lengths of one-fourth to one-half inch. These strips can be introduced and forced up with fine untempered instruments, readily uniting with the solution in the canal, and completely filling the root. In teeth having straight or nearly straight roots this is very easy, and it is my experience that it is the surest method in any case, as the solution can be forced into the most tortuous canals, and will fill them completely.

I have used oxide of zinc with creosote, following it and crowding it up the canal with phosphate of zinc rolled, as in the case of the gutta percha. Before that I used cotton saturated with carbolic acid at the apex, packing gutta percha or phosphate of zinc in the canal. I have used cotton dipped in oxy-chloride of zinc, which hardened in the canal. I primarily taught, and for many years practiced the filling of canals, whenever practicable, with gold, but I have had better results and feel better pleased with the gutta percha method.

My preceptor, Dr. R. M. Stenlee, of New York City, whom I had hoped to see to-night, taught me one thing thoroughly, viz: "whatever is worth doing at all, is worth doing well," and to that I owe much of my success in the treatment of devitalized teeth. But sometimes the result is unsatisfactory; the abscess, like the ghost in Hamlet, will not down at the bidding. As soon as the tooth is closed it begins to trouble the patient. In such cases, experience, patience and care will bring about the desired result in time. My chief annoyance has been when the tooth presented for treatment has never given any trouble, but is unmistakably dead. I open it carefully and treat it tenderly, and perhaps in a day or two have the patient return with periostitis and inflammation, and the mischief to pay generally. This condition does not arise from forcing foreign matter through the foramen, because it frequently happens when nothing has been done save to open the pulp chamber without entering the canal at all. I have not yet been able to find the reason for this, although it annoys me excessively.

I desire to say regarding the first division of my subject—treatment of recently devitalized teeth— that I rarely destroy the pulp intentionally, and have not done so more than twice during the past year. I then employed the usual arsenical preparation. I invariably attempt the salvation of exposed pulps when there is a reasonable hope that I shall be successful. The pulp once dead, however, I believe in careful and persistent treatment, for I believe that no diseased tooth is beyond salvation if it be judiciously handled.

On motion of Dr. J. Allen Osmun, of Newark, a vote of thanks was tendered Dr. Palmer for the paper of the evening.

Dr. Palmer then exhibited some teeth prepared and filled by Dr. Watkins, of Montclair, which clearly showed how the pink gutta percha might penetrate the canal. He also exhibited a number of the broaches used, showing how readily they could be bent in any direction, winding floss silk upon them to demonstrate his method. He explained the manner of drawing the temper by simply imbedding the broaches in a quantity of asbestos, or sand, and after heating thoroughly, allowing them to cool gradually. A very small quantity of asbestos between two pieces of sheet iron will suffice. The broaches may be readily obtained of any dealer in jeweler's materials.

Dr. W. Pinney, of Newark, narrated a case in practice, and gave a summary of his experience, corroborating much of Dr. Palmer's experience.

*Dr. C. F. W. Bodecker*, of New York: Dr. Palmer in his paper asks for information about the treatment of such devitalized teeth as have not given the patient any trouble prior to theopening of the pulp chamber, but which, as soon as this is done, even if no attempt has been made to enter the root canal, develop an alveolar abscess.

In some cases we can open the pulp chamber of a devitalized tooth, clean out the canal and fill it at once, and no trouble whatsoever will follow. In these instances the end of the root is encysted and almost any kind of filling, or even no filling at all, will produce a satisfactory result. In another class of cases; such as those of which Dr. Palmer speaks, we find no encystment, and consequently there is a communication of the pulp canal with the peripheral tissues about the end of the root. These are exceedingly troublesome to treat in the old manner, that is, without proper disinfectants, introduced into the tooth before the pulp cavity is quite opened; and here, in the majority of cases, an alveolar abscess is the result, which, I believe, is caused by contact with the air. For about eighteen months I have been very successful in such cases, and quite a number of my professional friends, who have pursued the same line of treatment, have met with the same results. My proceeding is as follows: I drill a hole into the tooth or filling, toward the pulp chamber, until it very nearly reaches it. I fill this drill hole with a saturated solution of iodoform in ether (about ʒi of iodoform to ℥i of sulphuric ether) and very quickly, before the ether is evaporated, pierce the remaining septum of the pulp chamber. I then fill the drill hole with a piece of cotton saturated with the iodoform solution, and temporarily seal it. This plug I allow to remain from three to five days before I attempt to clean out either the root canal or the pulp chamber. After this time has elapsed, I remove the temporary plug together with the cotton, render the pulp cavity accessible by making the entrance to the root canal as straight as possible without interfering with the strength of the tooth. I then clean out the pulp chamber by cutting away all superfluous dentine, and thoroughly syringe it out with water. I apply the rubber dam, dry out the cavity with bibulous paper, and introduce one or two drops of a twenty per cent solution of chloride of zinc. After waiting a few minutes I begin to clean out the root canals, either with Donnelson's nerve extractors, or the smooth broaches referred to by Dr. Palmer. These non-barbed broaches I have used for many years, and I know of no others that I like so well. In canals which are very narrow I renew the application of chloride of zinc once or twice, and then thoroughly wash out the root canal with absolute alcohol, using cotton wound around a smooth broach, until the cotton comes out unstained. I then apply a little of the saturated solution of iodoform in ether, which, by means of a smooth broach, I pump into the root canal. The next step is the filling of the canal, for which there is probably no better material nor method than that mentioned by Dr. Palmer in his paper.

At the meeting of the American Dental Association, held in New York City about two years ago, Dr. McKellops, of St. Louis, read a very able paper on the use of gutta percha in root fillings, which, it seems, has made a very deep impression upon many other dental practitioners besides myself. However favorable this material and method appeared to me then, I could not make up my mind to adopt it without first experimenting with it out of the mouth. I took two roots of lower bicuspids which had just been extracted; I removed everything out of the canals by means of a burr, after which I filled one of these roots with a solution of gutta percha in chloroform, followed by hard gutta percha without any further delay, but the other root canal I thoroughly washed out with absolute alcohol, and dried with bibulous paper and the hot air syringe, before the gutta percha was introduced. After two or three days, when the filling material was quite hard, I split both of these roots and found by placing them under the microscope that where I had used absolute alcohol for the dehydration of the dentine previous to the introduction of the filling material, the dentinal canaliculi were filled a little distance with gutta percha, whereas in the other root I could not see any gutta percha at all in the tubules. This result induced me to lay aside all other filling materials for filling root canals. I had considered gold the worst material for filling root canals, and up to this moment my experience is the same.

Dr. Palmer states in his paper that sometimes we may extend the treatment of pulpless teeth too long. I believe that when the pulp canal is thoroughly cleaned it should be filled immediately, unless a latent abscess is present. A failure in the result of the treatment of the teeth as above described is extremely rare, except where roots are inaccessible or curved.

*Dr. G. C. Brown.*—About three months ago I met with a very interesting case which was referred to me by Dr. Palmer, of New Brunswick. The patient had been under his care for about a year, when she removed to Elizabeth, and was thus placed beyond the reach of his treatment.

The trouble is of long standing and arises from the right superior canine, a dead tooth which has been filled a number of times, but from which in each instance the filling soon had to be removed because of the soreness and inflammation which supervened. I found a large pulp canal in an apparently healthy condition. I placed in it a dressing of iodoform and closely sealed the cavity. The next day the patient returned with the complaint that she had suffered excruciating agonies with the tooth, and, finally, to obtain relief, had removed the dressing. The cessation of pain was instantaneous. I made a very careful examination and after considerable difficulty succeeded in bringing to light a piece of a wooden toothpick about a quarter of an inch in length. The removal of this was followed by considerable hemorrhage. Thinking I had discovered the source of the trouble I sealed the cavity up again, with precisely the same result as before, and in this condition it has remained for three months. I cannot insert in the canal a small bit of cotton without inducing an excessive pain.

*Dr. C. F. W. Bodecker.*—When an alveolar abscess either by neglect or overtreatment has become chronic, it becomes very refractory to treatment unless handled energetically. In the majority of these cases necrosis is one of the inevitable results, and we can effect no cure until the sequestrum, however small it may be, has been removed, either by nature, by encystment, or by mechanical or chemical means. The case referred to by Drs. Palmer and Brown is one of this class, and in my opinion has been treated too long. From the description of the case I should advise the thorough cleaning, disinfection, and filling of the root canal; and if any more trouble arises, an incision should be made into the gum just above the end of the root. Through this opening everything necessary can be done. If a piece of necrosed bone is present it may be removed when loose, by a pair of fine tweezers. If no separation has as yet taken place, the removal of it by the burr may be justifiable. If a latent abscess is the cause of the disturbance it may be destroyed by the burr, the galvano cautery, nitric acid, or chloride zinc, either of which will produce the desired result.

*Dr. J. Allen Osmun.*—I would like to ask Dr. Bodecker how he would account for the following case: A gentleman presented himself with a right lower molar, which had been treated by another dentist previous to coming into my hands. Creosote, carbolic acid, salycilic acid, and iodoform had been used at different times with the following results. When all pain was under control the tooth was filled temporarily, and remained quiet from six weeks to three months, and then the pain would recur, although I am confident that I went to the apex of the root.

*Dr. Bodecker.*—I believe if the root canals had been filled permanently at the first or second sitting, the pain would not have recurred.

*Dr. M. L. Rhein.*—Mr. President, I would like to say a few words regarding this joint patient of Drs. Palmer and Brown, and in doing so I may throw a little light on the case in question. I can more easily illustrate what I want to say by citing a somewhat similar case, and giving my treatment thereof.

In April, 1882, Mr. H., aged about 33, came to me to have his mouth put in a healthy condition. By May 1st, I reached the superior right canine. The tooth had been, as he expressed it, "nothing but a shell for years," there being very extensive decay on the mesial-approximate and palatine, and on the distal-approximate and palatine surfaces. The pulp had evidently been dead for a long time. He had no remembrance of ever experiencing pain in the tooth, and there had been no alveolar trouble, as the extensive opening had given full vent to the sewerage present. I prepared the root and found that the decay had so softened the tissues that there was a broad opening to the gum, and the end of the root was necrosed. After a thorough cleansing of the canal, I put in my customary disinfectant, creasote (I was not then using iodoform), and hermetically sealed the cavity. The next morning the patient returned with a sore tooth, and I changed my application. He returned shortly again with the tooth in a worse condition. I carefully syringed out the canal, and put in a compound iodoform application. This was at 6 P. M. The next morning at 8 he returned with his face considerably enlarged, and suffering very much. I removed all applications and allowed him to go with the cavity open until the inflammation had subsided. I had determined to cease treating that root in that manner, but to reach the seat of the trouble through the process. On May 14th I made an opening through the gum as follows. After a local application of chloroform, I plunged the bistoury in the gum to the process, directly over the extremity of the root. Then with the aid of a tampon dipped in aromatic sulphuric acid, the opening was soon enlarged sufficiently to permit me to easily penetrate the process with a very large sized drill, the whole operation giving the patient very little pain. I then used the cross-cut burr to remove the diseased part of the root. This portion of the operation was somewhat more unsatisfactory, as it was impossible to accurately know how much tissue I was removing. Contrary to Dr. Atkinson's teaching, I left the canal open so as to enable me more efficiently to remove the debris of the root by syringing through the tooth. Through the opening in the process I inserted a tent of slippery elm, so as to prevent union taking place. Two days later, on making examination of the end of the root, I still found slight necrotic symptoms. Instead of again resorting to the burr I made use of pure nitric acid. Having properly shaped a pine stick, I dipped it in the fuming acid and applied it directly to the end of the root. This, though taking but a moment, caused severe pain. Another plug of slippery elm was inserted so that the wound might heal from the bottom by granulations. Two days later I filled the root with gutta percha almost to the neck of the tooth, and sealed up the remaining portion of the cavity with oxy-phosphate of zinc. The plugs of slippery elm were removed almost every day, and smaller ones inserted. By June 4th, the opening in the gum had entirely healed, it being three weeks after the operation, and the tooth was in a perfectly comfortable condition. On July 21st, after cutting out a sufficient amount of the oxy-phosphate, I filled the tooth with gold, inserting eleven sheets of No. 4 cohesive foil, with the aid of the electro-magnetic mallet. At this time there was new formation of bone in the process. Not the slightest irritation followed the restoration of the contour.

This case was brought fresh to my memory by the patient calling to see me a few days ago for his yearly examination. At that time I found the tooth just as I left it, with the exception of a slight amount of hypertrophy over the seat of the operation, caused by the formation of new bone. I have detailed this case because I think a somewhat similar treatment is needed in the case mentioned. In conclusion, I would add that I coincide with Dr. Bodecker when he states that the trouble with most of these uncurable abscesses is that they are over-treated. Clean your roots thoroughly, disinfect them with iodoform, fill them with gutta percha, using chloroform to make them perfect. If now there should remain a diseased condition below the apex, reach the seat of the trouble by penetrating the process over the point of the apex of the root.

*Dr. S. C. Watkins.*—Dr. Palmer in his paper refers to my method of treating and filling roots of teeth, which I described at the April meeting of this Society. When a case is presented to me with a diseased pulp I apply arsenic, seal the cavity tight, and dismiss the patient for four or five days. When this time has expired, I remove the pulp as thoroughly as possible. In some cases all of it can be taken out; in others, such as the buccal roots of the superior molars, the mesial roots of the lower molars, all of the wisdom teeth, and very often the first bicuspids when the roots are slender and crooked and the nerve canal exceedingly small, I assert that the pulp cannot be all taken out to the apicial foramen, by any one. When the pulp is removed as nearly as possible, and the nerve canal cleansed with alcohol, either by injections with the hyperdermic syringe or a bit of cotton twisted upon a broach, and disinfected with carbolic acid, the iodoform is introduced in the form of a paste. It is easily pumped into the canal by a few fibres of cotton entwined about a nerve broach. Then I use bibulous paper to wipe out the cavity and take up the glycerine and carbolic acid. When all this is completed, if the medicine has been thoroughly carried to the point of the root, the canal is ready for filling, which I complete at the same sitting, in the following way:

Dissolve pink gutta percha in chloroform. Wind a few fibres of cotton on a stiff broach and dip it in the solution. If the tooth is on the lower jaw, throw the chin well down; if it is an upper tooth, throw the head back and chin up, as far as possible. Then proceed to pump the gutta percha into the canal. Keep the entire cavity filled with the mixture, so that each time the piston is drawn and pressed into the canal, it will force some of the gutta percha ahead of it until the patient gives indications of pain, which is good evidence that the canal is full. Warm a piece of gutta percha and form it into a cone, and force it into the end of the canal; or a bit of cotton dipped into the solution of gutta percha and forced into the canal will drive it ahead, until you can be positive that you have the canal thoroughly filled, no matter how crooked or ill-shaped the roots may be, or how many there are of them. If you have not been able to get all the nerve out you need not lie awake nights worrying about it, for if thoroughly filled in this way it makes but little difference, because the iodoform, if carried through the apicial foramen, will disinfect the parts thoroughly, and prevent further decomposition. The liquid gutta percha will flow around any particles of remaining tissue and seal them hermetically. Even the ends of the canaliculi will be filled. If some of the filling should pass through the apicial foramen into the tissues beyond, they will tolerate it better than any other material.

In cases where the nerve has been dead a long time and an abscess has formed and opened through the gum, I clean the canal, treat and fill just as I have described, all at one sitting. If the abscess has no opening except through the tooth, I cleanse thoroughly with alcohol and carbolic acid, or a weak solution of chloride of zinc, then pump iodoform through the canal into the abscess at the end of the root, and close the cavity for three or four days, at the end of which time if there is still pus, or a disagreeable odor arising from the abscess or decayed matter, the same treatment is repeated. If it is entirely aseptic I fill at once.

I have used iodoform for ten months without a single failure, or any pain which lasted longer than twenty-four hours, and I have very seldom been obliged to make more than two or three applications in order to secure the desired result.

When the pulps of the temporary teeth are exposed, I make a bowl around them of gutta percha and fill it with iodoform paste, and fill the remainder of the cavity with whatever seems suitable for the occasion. When the pulp is dead I clean out the cavity, and the dead pulp and decayed matter from the pulp chamber, place therein some iodoform and fill with oxy-phosphate, or gutta percha. My experience has taught me that it is a perfect cure, and it may all be completed at one sitting.

The prescription for the iodoform I took from Dr. Bodecker's paper on the minute anatomy of the teeth, which he read before the Odontological Society, in March, 1882. It is as follows:

℞ Iodoform,
Kaoline, aa—ʒ ss.
Acid Carbolic, gtt viii.
Glycerine to form a paste.
Add Oil of Peppermint, gtt x.

*Dr. H. W. F. Buttner.*—At the last meeting of the New Jersey Central Dental Association in Hoboken, January, 1883, Dr. James G. Palmer, of New Brunswick, asked my opinion as to the practicability of setting artificial crowns on natural roots containing a live pulp. Although I had made very few experiments in this direction, I assured him that I was thoroughly convinced of the practicability of an operation of that kind, and would perform it at the earliest opportunity. Dr. Palmer then requested me to set a left upper central crown on a root with a live pulp. The following is a concise description of the case : A young man, about 22 years of age, had lost half of his left upper central incisor by a blow. Although very near, the pulp was not exposed. The patient had the lost part artistically restored with gold, and the anchorage made as strong as the case would admit. The acclusion being unfavorable the apex soon gave way under the pressure of mastication. The lost part was again restored, with a view to increased strength, and although this operation was seemingly a perfect success, the patient and his friends were dissatisfied because of the large amount of gold displayed in the mouth. Unfortunately the tooth was again broken by an accidental fall. The patient now came under my care, and the case presented a rather unfavorable appearance for the application of my method. If the stump had been shorter, a single trephine without a centre pin, would have sufficed to cut a circular shoulder, which would have been sufficient to sustain a circular gold cap without a central pin or pivot. But in this case the stump was too long, and another course had to be pursued. I constructed an especial instrument, adhering strictly to my trephining system. It presented the appearance of two trephines, a small one inside of a larger one.

The former was intended to turn a circular shoulder on the extreme part of the stump, about $\frac{3}{32}$ of an inch deep. The latter turned a circular shoulder on the upper part of the stump, a little under the gum. In this way I obtained a double shoulder, one above the other, without in the least endangering the vitality of the pulp. An impression was taken with plaster of paris, and a model cast. Although the latter showed the relation of prepared stump and other parts clearly, I found it necessary to use on the model, the same instrument I had used for the preparation of the stump in the mouth, that any roughness of plaster and slight imperfections of the cast might be removed. A gold cap had been prepared, composed of two sizes, a smaller and a larger. The former was intended to encircle the lower part of the stump, the larger the upper part. Both had a depth of $\frac{3}{32}$ of an inch, and were firmly united by solder. Both these caps were smaller in diameter than the prepared stump, in order to obtain a perfect joint without cement or other plastic filling material. The gold cap was pressed upon the stump on the plaster model, a plain plate tooth, ground concave on the inner surface and backed with platinum foil, was accurately fitted against the cap in line with the adjoining teeth, then temporarily fastened to the cap with resin, both parts withdrawn from the model, invested together in plaster and sand, and soldered with eighteen carat gold solder, filling out the palatal contour to the natural size. After finishing the cap with porcelain face, the combined parts were driven on the stump by a few blows with a mallet.

Six months have passed by since this operation was performed, and at the June meeting of the New Jersey Central Dental Association I was informed by Dr. James G. Palmer, who has had the opportunity to watch the case, that no trouble whatever has ensued, and that the patient considers it equal to any of his sound teeth. The pulp seems to thrive quite comfortably under the gold cap, and has caused no inconvenience whatever. The attachment is mathematically perfect, and is less liable to fail than the most perfect gold filling, and the root is protected from decay by the gold cap encircling it at the neck.

*Dr. C. A. Timme* exhibited some very ingenious appliances, invented by Dr. R. Telschow, of Berlin, namely: A hydraulic press, used for stamping metal plates; a very small furnace for backing continuous gum, which is heated by ordinary coal gas; a pneumatic mallet driven by a pump, which can be applied to every dental engine ; and a mouth illuminating apparatus. (Dr. Timme will give a clinic with these appliances, at the State Society meeting, to be held at Asbury Park, July 18th).

On motion, the Society adjourned to meet in June, at Dr. W. Pinney's, 72 Park Pl., Newark, N. J.

JAMES G. PALMER,
*Secretary.*

## Figures and Tables

**Figure f1:**
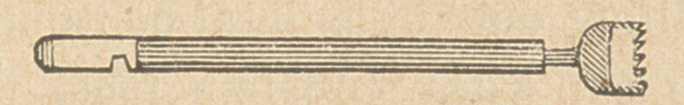


**Figure f2:**
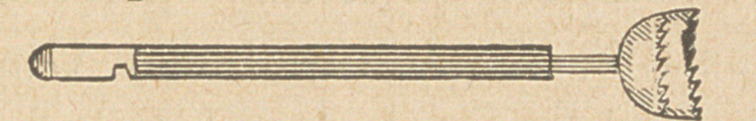


**Figure f3:**
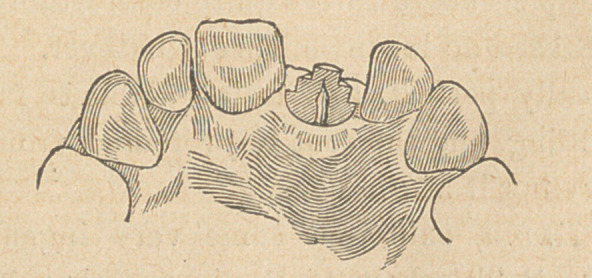


**Figure f4:**